# Potential disease transmission from wild geese and swans to livestock, poultry and humans: a review of the scientific literature from a One Health perspective

**DOI:** 10.1080/20008686.2017.1300450

**Published:** 2017-04-10

**Authors:** Johan Elmberg, Charlotte Berg, Henrik Lerner, Jonas Waldenström, Rebecca Hessel

**Affiliations:** ^a^ Division of Natural Sciences, Kristianstad University, Kristianstad, Sweden; ^b^ Department of Animal Environment and Health, SLU Swedish University of Agricultural Sciences, Skara, Sweden; ^c^ Department of Health Care Sciences, Ersta Sköndal Bräcke University College, Stockholm, Sweden; ^d^ Centre for Ecology and Evolution in Microbial Model Systems, Linneaus University, Kalmar, Sweden

**Keywords:** Antibiotic resistance, bacteria, human-animal-ecosystem interface, infection, parasites, pathogens, virus, waterfowl, wildfowl, zoonoses

## Abstract

There are more herbivorous waterfowl (swans and geese) close to humans, livestock and poultry than ever before. This creates widespread conflict with agriculture and other human interests, but also debate about the role of swans and geese as potential vectors of disease of relevance for human and animal health. Using a One Health perspective, we provide the first comprehensive review of the scientific literature about the most relevant viral, bacterial, and unicellular pathogens occurring in wild geese and swans. Research thus far suggests that these birds may play a role in transmission of avian influenza virus, *Salmonella, Campylobacter*, and antibiotic resistance. On the other hand, at present there is no evidence that geese and swans play a role in transmission of Newcastle disease, duck plague, West Nile virus, *Vibrio, Yersinia, Clostridium, Chlamydophila*, and *Borrelia*. Finally, based on present knowledge it is not possible to say if geese and swans play a role in transmission of *Escherichia coli, Pasteurella, Helicobacter, Brachyspira, Cryptosporidium, Giardia*, and Microsporidia. This is largely due to changes in classification and taxonomy, rapid development of identification methods and lack of knowledge about host specificity. Previous research tends to overrate the role of geese and swans as disease vectors; we do not find any evidence that they are significant transmitters to humans or livestock of any of the pathogens considered in this review. Nevertheless, it is wise to keep poultry and livestock separated from small volume waters used by many wild waterfowl, but there is no need to discourage livestock grazing in nature reserves or pastures where geese and swans are present. Under some circumstances it is warranted to discourage swans and geese from using wastewater ponds, drinking water reservoirs, and public beaches. Intensified screening of swans and geese for AIV, West Nile virus and anatid herpesvirus is warranted.

## Introduction

Some populations of geese and swans in Europe and North America have undergone dramatic growth during recent decades and they are now larger than any time in known history.[1,2] At the same time, outside the breeding season these birds have increasingly abandoned their natural foraging habitats in favour of croplands, meadows and turfs.[3] This and a generally reduced level of fearfulness have resulted in there now being more geese, close to more people, than ever before over large and densely populated areas in the Northern Hemisphere. This, in turn, has sparked conflicts with respect to crop damage, bird strikes at airports, fouling of drinking and recreational waters, eutrophication of wetlands, and degradation of natural vegetation. Although there are well-described local cases for most of these conflicts, their prevalence and consequences over larger spatial and temporal scales have only recently been reviewed comprehensively and critically.[3–5]

A recurring issue in this context is geese and other waterfowl as sources of infections (e.g. [6]). This is true for agriculture and food production, but also for human health via transmission of zoonotic diseases.[7] Interestingly, this is a rather recent concern, illustrated by it not even being mentioned in the influential monograph *Man and Wildfowl* by Janet Kear that was published in 1990.[8] Still, such worries are understandable, as it is commonplace to observe large flocks of geese and swans grazing and defecating in pastures and in fields producing food for livestock and humans. This behaviour brings these birds physically close to livestock during parts of the year, sometimes also close to poultry. Another concern is that large goose flocks for prolonged periods roost on lakes and wetlands where livestock drink and humans extract drinking water and swim.[5] On top of this, most goose populations are highly mobile, on a daily as well as a seasonal basis, making them potential disease vectors at short and medium spatial scales. Most species and populations are migratory, in many cases performing long-distance migration from tundra and high boreal areas in N and NE Europe, to wintering areas in W and C Europe. Consequently, geese and swans comprise an excellent model to study aspects of zoonotic diseases and disease transmission between wild and domestic animals. In this review, we apply a One Health perspective to tackle this question, reflecting the multifaceted disciplines involved in the study of diseases shared by several species.

The aim of the One Health initiative is to create common ground for several disciplines in order to establish more holistic approaches to diseases shared by more than one species.[9] Several major organizations, such as the World Health Organization (WHO), Food and Agriculture Organization of the United Nations (FAO), and World Organisation for Animal Health (OIE), are proponents of One Health, and the central disciplines are human medicine, veterinary medicine, and biology.[10] The One Health approach in the present paper is to combine findings about diseases and disease transmission in veterinary and human medicine with insights from ecology and management of waterfowl.

The overall number of pathogens found in wildlife is staggering, and so is the scientific literature on the topic. Partly as a consequence of this, most textbooks and reviews provide information with insufficient detail for practical management of more restricted taxonomic groups and geographical areas (e.g. [7,11,12]), for example in goose management in Europe. Another challenge is the rapid development of disease surveillance technologies, such as sequence and genome-based methods for detection and characterization of pathogenic microorganisms. Importantly, these methods open new avenues to differentiate between strains of pathogens, and thus to understand disease dynamics and effects better. Such information needs to be condensed and synthesized and put in more lucid writing before it can be used by managers and decision-makers.

Rapidly increasing goose numbers, goose–man conflicts, and increased knowledge about pathogens together call for an up-to-date review of the role of geese and swans as reservoirs, spill-over hosts and vectors of pathogens of known or suspected zoonotic potential. Wild herbivorous waterfowl can act both as biological vectors, i.e. harbouring an active infection where the pathogen develops and multiplies, and as mechanical vectors (containers, dispersers), i.e. physically dispersing pathogens from one site to another without being essential to the life cycle of the pathogen. Here we provide a comprehensive review of the scientific literature on the likely risk of disease transmission from wild herbivorous geese and swans to livestock, poultry and humans. We also discuss the implications of these patterns and identify knowledge gaps for management of and research about these birds and their habitats.

## Methods

The taxonomic scope of this review is geese and swans, which are obligate herbivores. We initially included other widespread anatid herbivores (Eurasian wigeon *Anas penelope*, American wigeon *Anas americana*, gadwall *Anas strepera*), but found little relevant data for them, and they were subsequently omitted from analysis.

Topical delineations used are: (1) Type of transmission: We included zoonotic transmission (animal to humans) and transmission to commercial livestock and poultry (risk for them *per se* and for secondary zoonotic transmission). (2) Transmission pathways: For transmission between different animal species including birds we considered potential direct and indirect transmission (via faeces, water, food plants, etc.). From birds to humans, we considered transmission by aerosol (inhalation), water, and direct and indirect contact (also through faeces). Finally, we included transmission from wild birds to humans via poultry and livestock. (3) Types of pathogens: we considered virus, bacteria (including acquired antibiotic resistance), and unicellular endoparasites relevant for livestock, poultry (with a focus on laying hens, broilers, turkeys, ducks and geese) and/or humans (Table 1).

**Table 1.** Pathogens/diseases with zoonotic potential in geese and swans treated in the present review. A large number of additional zoonotic diseases exists in wild geese and swans, but have been omitted because they are rare, extralimital or, according to current knowledge, have limited zoonotic potential.

**Viral diseases**
Avian paramyxovirus /Newcastle disease
Anatid herpesvirus/duck plague
West Nile virus
Avian influenza virus/AI/AIV
**Bacterial diseases**
Gastrointestinal
*Salmonella*
*Escherichiacoli*
*Vibrio cholera*
*Pasteurella multocida*/avian cholera
*Campylobacter* – *Helicobacter*
*Yersinia*
*Clostridium botulinum*/avian botulism
*Brachyspira*
Respiratory
*Chlamydophila psittaci*/chlamydiosis, ornithosis or psittacosis
Systemic
*Borrelia*/Lyme disease and borreliosis
Antimicrobial resistance
**Unicellular endoparasites**
*Cryptosporidium*
*Giardia*
Microsporidium

The list of pathogens with zoonotic potential detected in geese and swans is very long. Therefore, we used the One Health perspective to reduce the number subsequently treated here (see Table 1). In other words, very rare or extralimital pathogens were omitted, as were those with limited zoonotic potential, or with limited relevance to spread to livestock in developed countries. This review excludes disease transmission originating from domestic poultry facilities to humans and other animals, as well as ectoparasites, fungal diseases, and disease transmitted via human consumption of goose and swan meat.

The geographic focus of this review is Iceland, Denmark, Norway, Finland, and Sweden, but we have considered papers from other countries in Europe, as well as from Canada, the USA and northeast Asia when relevant. This is motivated by the fact that most goose and swan species in the Northern Hemisphere have long flyways covering many countries and climate zones. In addition, several species in the focal area occur naturally also in North America and northeast Asia, so it would be unwise to disregard studies from those areas.

We used the databases Web of Science, Natural Sciences Collection and Google Scholar. The dates of last access were 12 October 2015, 12 October 2015, and 8 January 2016, respectively. In retrieved papers we performed a backwards search of the reference list for further relevant studies, and the same was done with these until no further relevant papers could be found.

## Results

### Viral diseases

Many viral diseases are known to occur in waterfowl (e.g. [13]), and several most certainly remain undetected. Four groups of viruses – paramyxovirus, anatid herpesvirus, West Nile virus, and avian influenza virus – are of particular interest for the present review (Table 1).

#### Avian paramyxovirus/Newcastle disease

Newcastle disease (also ‘avian pneumoencephalitis’ and ‘pseudofowl pest’) is one of the more prevalent and economically devastating viral infections in the poultry industry, and it is widely spread over the world and endemic to numerous countries.[14,15] There are 12 serotypes in the genus *Avulavirus* (avian paramyxoviruses, APMV) in the family *Paramyxoviridae*. Newcastle disease is caused by the avian paramyxovirus serotype 1 (APMV-1), which occurs in both highly virulent (velogenic) and low virulent (lentogenic) forms. Velogenic strains cause massive die-offs in poultry farms, but are rarely detected in nature, with only few outbreaks recorded, primarily in cormorants.[16] Outbreaks of Newcastle disease are not known in wild waterfowl, but wild swans and geese are important asymptotic hosts of the more benign lentogenic APMV-1 strains (e.g. [17]). APMV-1 does not constitute a threat to human health, but it can cause mild conjunctivitis and influenza-like symptoms, which have mainly been reported in people working with infected poultry.[18] The other 11 serotypes of APMV do not cause Newcastle disease *sensu stricta* and are not considered here, although some of them have been associated with disease in poultry and other captive birds.[17]

Virological studies of APMV-1 in waterfowl (thus also including ducks) typically report a prevalence between 0.5 and 2.5% based on virus isolation (reviewed in [11]), whereas serological (antibody-based) studies generally report higher frequency; in swans and geese frequently a seroprevalence of 40–60% (n=1014 [19], n=130 [20], n=858 [21]). Muzyka et al. [22] reported on virus isolation in ca. 1800 samples from Ukraine from all seasons (mute swan *Cygnus olor*, whooper swan *Cygnus cygnus*, greater white-fronted goose *Anser albifrons*, greylag goose *Anser anser*, and red-breasted goose *Branta ruficollis*), but were able to isolate APMV-1 only in two greater white-fronted geese sampled in winter. German studies based on serology report either negative results (greylag goose [23]), 6–8% seroprevalence in breeding Canada geese *Branta canadensis*,[24] 14% in Canada geese in fall,[25] and 45% in greater white-fronted geese in October.[20] The only studies from the Nordic countries did not find any APMV-1 antibodies in greylag geese (Norway, spring [26] and Finland, fall [27]). Most studies of Newcastle disease have been based on cloacal swab samples. However, already Vickers and Hanson [28] and Wobeser [11] pointed out that it is far from certain that this is the best method to detect the virus, and that studies based on faecal swabs most likely underestimate APMV-1 prevalence in wild waterfowl. Contributing to the uncertainties related to AMPV-1 estimation is the fact that waterfowl can be infected by several different viruses simultaneously; for instance, Wille et al. [29] presented results suggesting that the dynamics of AMPV-1 in mallards *Anas platyrhynchos* were affected by co-circulation of AIV and avian coronavirus.

As stated above, Newcastle disease is not of immediate human health concern, but the role of swans and geese as reservoir or spill-over hosts [21,30] and their potential to act as long-distance biological or mechanical vectors [22,25,31] of precursors to velogenic APMV-1 strains remains poorly understood, and warrants continued attention (*cf*. [32–34]) from a poultry health perspective. Research to date, however, indicates that wild swans and geese do not have any role in outbreaks of velogenic APMV-1 on poultry farms, and Wobeser [11] moreover concluded that no evidence exists that it is established in wild waterfowl. Nevertheless, to prevent transmission of avian paramyxoviruses either way, wild and captive birds should not be allowed to get in contact, and it is further advised that they do not share water for drinking and swimming. Recently, Ayala et al. [35] provided evidence that vaccine-derived Newcastle disease virus can spill over from poultry into wild bird populations, but at present it is unknown whether this has any relevance for waterfowl.

#### Anatid herpesvirus/duck plague

Duck plague is caused by the anatid herpesvirus 1, also called duck enteritis virus (DEV) (genus *Mardivirus*, family *Herpesviridae*).[36] It occurs over Europe, North America and Asia,[13] and has been documented in ducks, geese, swans, and coots.[13,37] It is one of the most lethal infections known in waterfowl, with massive die-offs recorded in wild mallard and Canada geese in North America. The disease was first described from Europe (the Netherlands, 1923), but virtually all outbreaks in Europe have been minor and restricted to domestic or semi-domestic waterfowl.[11] Humans do not get infected by the virus.

The infection is known to spread between farmed and wild birds, but its epidemiology remains enigmatic. Waterfowl seem to be the virus’s only reservoir between outbreaks.[11] The faecal–oral pathway is probably the most common infection route, but transmission from mother to young through the egg also occurs. To date very few studies have addressed its occurrence in Europe, as it is currently not perceived as a threat to the poultry industry. In eggs from Canada geese breeding in Germany, Bönner et al. [24] found an antibody seroprevalence of 13% in 2002 (*n* *=* 107), but none (0%) the year after (*n* *=* 181). A Polish survey 2006–2013 of liver tissue from mute swan, greylag goose, bean goose *Anser fabalis* and mallard showed presence of anatid herpesvirus in 73% of 131 sampled birds and isolated three viruses.[38] Collectively, these studies suggest that anatid herpesvirus 1 is widespread among asymptomatic waterfowl in Europe. Since duck plague is a potentially devastating disease and because the etiological agent likely occurs in swans and geese in the Nordic countries, it is warranted to better monitor its distribution in space and time. In order to prevent outbreaks, domestic poultry should not be allowed to get in direct contact with wild waterfowl, and it is further advised that they do not share water for drinking.

#### West Nile virus

Originally endemic to Africa, the Middle East and South-West Asia, the West Nile virus (WNV) (genus *Flavivirus* in the family *Flaviviridae*) has spread rapidly into temperate areas of North America and Europe during recent decades.[39] Although primarily an avian pathogen, it can also infect amphibians, reptiles, and mammals, including humans. Among birds, infections vary from asymptomatic to severe depending on species, where outbreaks with high fatality rates have been seen in raptors and passerines such as crows.[40] WNV is of great concern to human medicine, too, as it is neuroinvasive and may cause potentially fatal encephalitis. The WNV outbreak in the USA in 2012 caused at least 286 human deaths.[41] The virus is transmitted by ornithophilic mosquitoes during blood-meals. Passerine birds are the most important reservoirs and amplifying hosts of WNV.[42] Some studies suggest that birds, including waterfowl, may also be long-distance dispersers of the virus.[43,44] As reservoirs for local spread, though, present knowledge suggests that wild swans and geese are unimportant relative to passerines.[42] Nevertheless, following the WNV outbreak in New York City in 1999, domestic geese and Canada geese were among the birds showing the highest seropositivity rate;[45] however, it is uncertain whether geese develop sufficient viremia to permit transmission to new hosts.

Detecting viremic birds in nature is rare, and most studies rely on serology, either ELISA or virus neutralization assays. Seroprevalence for WNV is generally low in European studies of wild birds (1–10%, reviewed in [46]). The only WNV screening study of birds from the Nordic countries showed very low seroprevalence (0.1%) among the 104 sampled species, and no signs of exposure to WNV in the seven tested waterfowl species.[47] However, there are several reasons to include WNV in the present review. Firstly, WNV antibodies have been found in mute swans in Poland, Germany and Serbia.[46,48,49] Secondly, the virus has spread steadily northwards, a process likely exacerbated by climate warming.[50] Thirdly, several of the mosquitoes that are competent vectors for WNV are widespread and common in the Nordic countries (*Culex pipiens, Coquillettidia richiardii* and at least 14 other species), and other species are expanding northwards (e.g. *Aedes albopinctus* [51]). Fourthly, swans and geese occur mainly in wet habitats where also mosquitoes are common, increasing the risk of virus amplification and subsequent spread. Finally, small WNV outbreaks with 10–40% fatality rates in humans with neurological symptoms have occurred as close as Romania, Serbia and Russia.[46,52,53]

At present, WNV is not a health issue in the Nordic countries, which is also mirrored by the relative few studies conducted hitherto. As demonstrated for WNV in North America, however, the situation may change rapidly depending on host, vector or climatological factors. Consequently, initiating surveillance for WNV in wild birds including waterfowl is a wise means to monitor any change to this situation.

#### Avian influenza virus/AI/AIV

The avian influenza virus (AIV) (genus *Influenzavirus* in the family *Orthomyxoviridae*) is a genetically variable, single stranded RNA virus. It has a large host range, including mammals (and humans), but the largest diversity of virus subtypes and genetic lineages is found in birds. The natural reservoir of the virus is wild waterbirds, in particular dabbling ducks, but also to a lesser extent swans, geese, diving ducks, and shorebirds.[54,55] Naturally occurring AIV subtypes are almost exclusively classified as low pathogenic (LPAIV), which means that they do not cause significant disease either in their wild hosts or in gallinaceous poultry. In dabbling ducks, LPAIV show an annual pattern of low prevalence in spring and summer, and a marked peak in autumn when prevalence may reach high values (20–30%) in the Northern Hemisphere.[55,56] Less is known about LPAIV in other waterbirds, but geese and swans are infected with LPAIV, though at a general lower prevalence rate.[54]

Some strains have the capacity to transmit from waterfowl to gallinaceous poultry, most notably the H5, H7 and H9 subtypes. In gallinaceous poultry, LPAIV can mutate into a highly pathogenic form (HPAIV), which causes devastating disease. The increased virulence is largely determined by mutations at the receptor binding site of the haemagglutinin molecule, leading to systemic, rather than localized infection. A driving force for this switch is a high mutation rate combined with high densities of immuno-naive hosts. Once established in a poultry facility, HPAIV is highly contagious and may develop into local, regional or even intercontinental epizootics in poultry.[54,57]

AIV can also transmit to mammals and humans, but as birds and mammals differ in which receptor types that are predominately expressed on cells, and in which tissue types the receptors are found,[58,59] the capacity of an AIV to establish and maintain itself in mammal hosts is limited. However, once an AIV has adapted to a mammalian host it may spread and become epidemic in it. It is important to note that AIVs can affect humans and animals in two ways: either via direct transmission (such as HPAI H5N1, which although it is rare as a spill-over infection is associated with high mortality in humans) or via contributing novel antigenic properties through reassortment of avian and mammal AIVs in co-infected hosts (such as the pig, which expresses both avian and mammal receptors). Events of the latter type may in the worst case spark new flu pandemics.

Up to recent years, the conventional wisdom has been that HPAIVs are restricted to poultry, and do not circulate widely among wild birds. This notion has been challenged lately, partly as a result of the ongoing epizootic of HPAI H5N1 and associated reassortant viruses in Eurasia, Africa and North America (e.g. [60]). The severity of HPAIV infection varies among wild birds, with systemic infection and high mortality in most species, and subclinical to asymptomatic infections in some waterfowl, particularly in dabbling ducks. This change in epidemiology is not fully understood, but contributing factors may include partial protection from previous LPAIV infections and adaptations of the virus associated with reduced virulence. In any case, migratory waterfowl may act as reservoirs and of HPAIV and contribute to dispersal via migration (e.g. [61–63]). Health concerns for poultry, livestock and humans are the main reason for the huge body of research on AIVs, including their main avian hosts. The mallard and closely related dabbling ducks (American black duck *Anas rubripes*, northern pintail *Anas acuta*) are the model species in this research. This situation presents a challenge for the present review, as susceptibility to AIV varies considerably among waterfowl species. In a meta-analysis based on studies involving ca. 45,000 sampled waterfowl, Olsen et al. [54,Table 1] found that AIV prevalence is 10 times higher in dabbling ducks such as mallard, American black duck and northern pintail, than it is in geese and swans. The literature on AIV in swans and geese is relatively sparse, but it appears that swans and at least some goose species face high mortality when exposed to HPAIV.[54,64–67] For example, a single outbreak in China reduced the global population of wild bar-headed geese *Anser indicus* by 10%.[68] If this is a general pattern, swans and geese rather become poor reservoirs and vectors of HPAIV (*cf*. [61]), as very ill or dead birds do not fly far.

Studies of LPAIV in swans and geese generally demonstrate very low prevalence of infection, often in the range of 0–3% in fresh faeces (Canada goose;[6] mute swan;[21,69] several species [70–72]). On the other hand, data on seroprevalence suggest that many swans and geese eventually do become exposed to LPAIV: 0–14% in Canada goose,[6,24,73] 45% in mute swan,[21] 63% in pink-footed goose *Anser brachyrhynchus*,[74] and >95% in Emperor goose *Chen canagica*.[72] Lambrecht et al. [69] studied 520 mute swans in Belgium 2007–2010 and found that seroprevalence varied with age, being higher in adults than in juveniles (54 and 16%, respectively), and furthermore that seroprevalence rate varied with sampling season and whether birds utilized stagnant or flowing waterbodies. How LPAIV infection affects swans and geese in the wild is, however, still poorly understood. Van Gils and co-workers [75] found that infected Bewick’s swans *Cygnus columbianus bewickii* reduced feeding and delayed spring migration departure, but this study was based on two birds only. Conversely, in a study of thousands of greater white-fronted geese, Kleijn et al. [76] did not find any difference in movement behaviour between infected and non-infected birds. These patterns have been interpreted very differently; some researchers argue that geese and swans are important to AIV dynamics in general. Others, for example Harris et al. [6], argue that Canada geese play only a minor, if any, role as a reservoir for LPAIV in nature. Another argument why geese may be less important is the limited persistence of AIV in faeces and low viral shedding following experimental infection.

Although the importance of dabbling ducks as hosts, reservoirs and short-distance dispersers of AIV is undisputed, also in the Nordic countries,[70] the role of swans and geese in these processes is still poorly understood (e.g. [77]). For example, virus prevalence ([72]; data from four goose species in Alaska) as well as seroprevalence ([74]; pink-footed goose) have been found to be higher in spring than in fall, which is opposite to the seasonal pattern in dabbling ducks. Although this suggests that AIV dynamics in geese and swans may differ significantly from those in ducks, caution is warranted as the former findings are based on a much smaller body of literature, and may be obscured by other factors, such as the seroconversion rate and maintenance of detectable serum antibodies. Furthermore, previous exposure to LPAIV may affect susceptibility and disease severity of HPAIV infections, as has been noted in both experimental [78–80] and field-based investigations.[81,82] Hence, increased monitoring of AIV in geese and swans is called for, not least since they occur close to agriculture (crops, livestock, and poultry) and humans. Keeping wild and farmed birds separate from each other is a wise preventive measure for AIV. Regardless, HPAIV in wild geese and swans is not perceived as a health concern for humans, unless one is in direct contact with infected birds.

### Bacterial diseases

Several pathogenic bacteria can be found in geese and swans. We have grouped them according to the organ system they chiefly affect: gastrointestinal, respiratory, and circulatory (Table 1). Antibiotic resistance has been given a separate section.

#### Gastrointestinal

##### Salmonella

The genus *Salmonella* is distributed worldwide and due to its predominantly clonal population structure it displays a large number of serovars, some of which have the capacity to cause intestinal disease in humans and animals, including birds, by the faecal–oral transmission route.[83] There is often limited host species specificity, and the disease is considered zoonotic (typically acquired by contaminated food). In wild birds, salmonellosis is known from a wide variety of species, but mainly known to cause severe disease and mortality from septicaemia in small passerines,[84,85] whilst many other species carry asymptomatic infection (e.g. gulls [86,87]). Because of its relevance to the poultry industry, including risk of alimentary infections in humans, quite a large number of studies has been carried out on its epidemiology in wild birds. Further, there are recommendations within the poultry industry to avoid or minimize contact between wild birds and domestic poultry. In particular, the presence of infected gulls and passerines has been suggested as a risk factor for salmonellosis in domestic animals.[85] Wild birds are not considered a main source of infection for livestock, though, instead feed contamination and recycling among farm animals are often the source.[83]

A link to human activities has been suggested, and when *S. typhimurium* bacteria were found in mute swans in the UK the authors concluded that the birds had come from a contaminated environment related to human sewage overflow and dirty surface water.[88] Furthermore, *Salmonella* has been isolated from droppings of Canada geese (0–8% except one site where the prevalence was 20%, *n* *=* 50) in UK parklands, and it has been shown that *Salmonella* bacteria in Canada goose droppings can multiply and survive for up to one month in this environment.[89] However, a Norwegian study of waterfowl shot in mainly densely populated areas reported very low prevalence of *Salmonella* positive samples (1.4% of carcasses, but all droppings negative, *n* *=* 182 [90]), and another Norwegian study found only one infected Canada goose in a summarized post-mortem covering the years 1969–2000 (*n* *=* 40).[91] Similarly, Lillehaug and co-workers [26] found just one greylag goose positive for *S. diarizona* (*n* *=* 100), which is in accordance with a Swedish study of faecal samples from 200 Canada geese shot during the hunting season, in which none turned out positive for *Salmonella* spp.[92] Similarly, a German study of wintering brent geese *Branta bernicla*, barnacle geese *Branta leucopsis*, greylag geese, greater white-fronted geese, pink-footed geese, and bean geese, found no *Salmonella* positive faecal samples at all.[93] Neither did a German study of Canada goose eggs (*n* *=* 289) find any *Salmonella* spp.[24]

Predominantly negative results have been obtained in US studies of Canada geese, where 0% (*n* *=* 318) and 0.01% (*n* *=* 449) respectively of the samples from non-migratory Canada geese were positive for *Salmonella* spp.[94,95] Likewise, in a New Zealand study of Canada geese (*n* *=* 80) and black swans *Cygnus atratus* (*n =* 80) all samples were negative.[96]

Refsum alone and together with co-workers [85,90] proposed that the importance of waterfowl in spreading *Salmonella* bacteria is limited, except for birds resident in areas highly contaminated by human waste or domestic animal manure. However, Gorham and Lee [5] rather emphasized the uncertainty of assessments of potential risks, especially in relation to Canada geese. There are no studies directly linking outbreaks of salmonellosis in humans, livestock or domestic poultry to the presence of swans and geese or their faeces. However, the absence of such findings may partly be a result of existing biosecurity routines, and hence it is nevertheless wise to apply precautionary principles and ensure that domestic poultry do not get in contact with, or share pasture or water access with, wild waterfowl. The absence of reports about outbreaks in ruminants linked to waterfowl indicates that in practice the presence of such birds on grasslands and pasture grounds, which is a common phenomenon in many regions, may not constitute a major risk factor for *Salmonella* infection in livestock. Furthermore, the presence of geese in urban parks and on beaches does not, based on the reasoning above, appear to constitute any major human health risk with respect to *Salmonella*.

##### Escherichia coli

*Escherichia coli* (*E. coli*) is a bacterium that can normally be found in the intestinal tract of humans and animals, including birds. Most strains are harmless and often considered part of the normal gut flora in vertebrates. For example, mute swans can carry *E.coli* bacteria (60%; *n* *=* 15) without showing any symptoms of disease.[97]

However, some *E. coli* strains have pathogenic properties, often plasmid mediated, and can cause diarrhoea and systemic illness in humans and/or animals, i.e. they are zoonotic. *E. coli* can be spread by food and water contamination. From a human health perspective, only strains carrying human virulence factors are of importance.[98] With respect to domestic animal health, a slightly wider range of virulent strains can be of relevance.

Migratory geese frequently fly between rural and urban areas. In rural areas where livestock concentration and the prevalence of infection with virulent strains of *E. coli* are both high, geese may be exposed to such bacteria and become infected, and later disperse the strain. Wherever faecal contamination occurs, *E. coli* may be present.

Prevalence of *E. coli* in Canada goose droppings in parks in the USA varied considerably among seasons (as low as 2% in the cold season, and up to 94% in the warmest months; *n* *=* 397), and further the proportion of *E. coli* with human virulence factors was low (2%).[99] The authors concluded that Canada goose faeces do not pose a significant risk to human health, but that it is nevertheless wise to minimize contact with faecal material and to remove shoes before entering homes. It has been shown that Canada geese can be a relevant source of *E.coli* on beaches in North America [100,101] and that a low number of these birds carry enteropathogenic *E. coli* (8%, *n* *=* 90),[102] but this has not been linked to disease in humans. In a study of faecal samples from 200 Canada geese shot in Sweden all were negative for VTEC 0157, a strain responsible for potentially severe disease in humans.[92] However, a study of black swans and Canada geese in New Zealand showed an *E. coli* prevalence of 94–95% (regardless of strain).[96] A British survey of 12 sites revealed a large variation in presence of *E. coli*, ranging from below 10% at some sites to 100% in others (*n* *=* 50).[89]

As water quality in lakes and rivers can be affected by high levels of coliform bacteria, it is highly relevant to try to establish the source of such faecal contamination. Hence, several studies have studied *E. coli* isolates from a host species perspective. Two Canadian studies showed that isolates were relatively host-specific and that *E. coli* strains from geese totally dominated in faecal material from geese (87.5% (*n* *=* 7) and 79.7% (*n* *=* 44) respectively [103,104]). A Dutch study of *E. coli* concentrations in faeces from geese and other birds on recreational waters indicated that gull faeces contained higher concentrations of *E. coli* than did goose faeces (greylag goose; *n* *=* 25) [105] and a study of recreational waters in the USA revealed that human faecal material was as common a source of contamination as was goose faecal material, especially after rainfall.[106] However, geese can also carry *E. coli* strains of human origin (12.5%), and humans and dairy cattle can carry strains of goose origin (2.9 and 14.3% respectively [103]. In a recent study, Kuczkowski and co-workers [107] reported both geographical and interspecific differences (mute swans (*n* *=* 37), greylag geese (*n =* 61) and Canada geese (*n =* 33) in Poland and the Netherlands, respectively) in the pathogenicity of the *E. coli* strains identified. An important question to ask is whether *E. coli* constitutes a normal part of the gastrointrestinal flora of geese and swans, or whether high prevalence of this bacterium in itself is a sign of transmission from anthropogenic sources.

In summary, it is difficult to draw any firm conclusions about the risk of transmission of *E. coli* from geese and swans to livestock or humans, other than that in most cases the *E. coli* found show low prevalence of human virulence factors and is hence not an important source of zoonotic infection. Nevertheless, direct human contact with goose and swan faeces should be avoided in general.

##### Vibrio cholerae

*Vibrio cholerae* is a zoonotic bacterium found in aquatic environments.[108,109] There are both pathogenic and non-pathogenic strains, the former producing a toxin that can cause very severe diarrhoea and vomiting in humans, even fatal dehydration in the absence of proper medical care. The main sources of infection are contaminated drinking water, but also consumption fish and shellfish. Animals including birds show no symptoms when infected.

*V. cholerae* has been reported in fresh faecal samples from Canada geese (6%; *n =* 16), Mute swans (67%; *n =* 3), and other aquatic birds in coastal areas in the USA where the bacterium is known to occur.[110] It has been hypothesized that aquatic birds can serve as vectors or reservoirs of *V. cholerae*, but very little is known about the potential significance of this. Another US study, focusing on inland waters, did not find any positive cloacal swab samples from Canada geese (*n =* 43), although some other aquatic bird species were positive.[111] *V. cholera* was also detected in water and faecal (*n =* 55) samples in a lake in a Japanese agricultural area well-known for its staging and migrating Greater white-fronted geese.[112] However, the limited information in the literature supports the conclusion that *V. cholera* from swans and geese is currently not a relevant risk in Europe.

##### Pasteurella multocida/avian cholera

*Pasteurella multocida* is the bacterium responsible for outbreaks of avian cholera, also known as fowl cholera in domestic poultry. *P. multocida* is zoonotic, but causes quite different diseases, with different denominations, in various bird and mammal species.[109,113] Humans are mainly infected via pet scratches and bites, leading to wound infections. Usually, fowl cholera is caused by a limited number of specific serotypes and is considered a disease relevant only to wild and domestic birds. In wild aquatic birds the disease is currently most prevalent in North America, and outbreaks are much less common in Europe.[114] The acute form of the disease is often fatal in birds. Affected birds show diarrhoea or are, in the case of waterfowl, often just found dead, whereas chronically infected birds can display symptoms in various organ systems.

Snow geese *Chen caerulescens* can be infected with the bacterium but survive,[115] although mortality in this species is high in some outbreaks, also during migration.[116] Outbreaks have also included Canada geese.[116] Greater white-fronted geese show a low prevalence of antibodies (< 5%, *n =* 590) at breeding sites in Alaska.[117] In the latter study the authors were not able to isolate *P. multocida* from any oral, nasal or cloacal swabs analysed (*n =* 1227), and they hence concluded that these geese were unlikely to be important carriers of the bacterium,[117] although it has been identified in samples from several goose and swan species.[114] Investigations in the USA have evaluated if wetlands *per se* can function as a primary reservoir for the bacterium between outbreaks, as they will inevitably become contaminated with *P. multocida* during an outbreak. However, this seems not to be the case, as no positive sediment samples (*n =* 440) were found by Samuel et al. [118]. It has been hypothesized that *P. multocida* occurs latently in healthy waterfowl acting as mechanical vectors,[116] and in the USA the snow goose has been proposed as a relevant carrier species.

In practice, *P. multocida* infections in geese and swans do not appear to pose any significant risk to humans or livestock. For waterfowl the disease is currently mainly an issue in North America, but it is nevertheless wise to keep waterfowl separated from domestic poultry also in other parts of the world.

##### Campylobacter – Helicobacter

Most *Campylobacter* species are adapted to the intestinal tract of animals, and several species are found in wild birds (including waterfowl), where they are considered commensals.[119] This is in stark contrast to humans, where campylobacters – especially *Campylobacter jejuni* and to a lesser extent *Campylobacter coli* and other species – cause gastrointestinal disease. In fact, campylobacterioisis is the most commonly reported cause of bacterial gastroenteritis in humans worldwide, with diarrhoea, abdominal pain and vomiting as main symptoms.[120] The main source of infection is consumption of contaminated water or meat, especially poultry meat, and other food products. The association with domestic poultry has prompted a large number of studies of *C. jejuni*.

Various species of *Campylobacter* have been isolated from faecal samples and cloacal swabs from apparently healthy geese in many different geographical locations, but it is not clear if waterfowl have a significant role in zoonotic spread.[119] One study implied an outbreak of *Campylobacter* infection in humans to be connected to pink-footed geese staging in the vicinity of a water reservoir (from which untreated drinking water was taken), but no faecal samples from the geese were analysed.[121] A German study of eggs (*n =* 289) from Canada geese did not find any *Campylobacter* at all in the embryonic tissue,[24] which was not surprising as vertical transmission of *Campylobacter* via eggs does not occur in any bird species, whereas a Swedish study of 200 Canada geese shot during the hunting season found 15% of the samples positive for *Campylobacter* spp.[92] Similar prevalence levels (12–23%) were reported from Barnacle goose faeces in Finland in summer (*n =* 924).[122]

Modern typing methods are necessary for establishing links between isolates of different origin.[119,123] For example, when looking at *Campylobacter* populations in wild geese and free-ranging poultry on the same farm, Colles et al. [124] found that although a large proportion of the Canada and greylag geese included did carry *C. jejuni* (50.2%, *n =* 331), these bacteria were from a genetically different population than the ones identified from free-ranging broilers sampled at the same location. Hence, the *Campylobacter* isolated from geese appeared host specific, and their contribution as a source of infection to humans and farm animals were most likely minor.[122,124] Similarly, the prevalence of *C. jejuni* in non-migratory Canada geese (*n =* 318) in the USA was reported as ranging from 5.0% to 16%, but the strain types from these geese were not previously encountered among human clinical cases or farm animals.[94] A Swedish study of migrating brent geese indicates that prevalence of *Campylobacter* may be rather low (one out of four sampled geese tested positive), and the authors stressed that it is unknown if this particular strain, and other isolates from the same study, are transmissible to humans or domestic animals.[125] In a study on wild birds and domestic cattle it was concluded that although these birds, mainly shorebirds and barnacle geese, shared a common environment during the grazing season, the different host species largely carried their own types of *Campylobacter*. From this, Waldenström et al. [126] drew the conclusion that between-species transmission is rare. This is in accordance with Llarena and co-workers,[122] who concluded that barnacle geese are probably an infrequent source of campylobacteriosis in humans, a conclusion which can most likely be extended also to other goose species.

*Helicobacter* is a group of enteric bacteria regarded as related to the *Campylobacter* genus, and are believed to have zoonotic properties.[127] Some are pathogens; e.g. *Helicobacter pylori* is known to cause gastritis in humans, and also *H. canadensis* has been linked to disease in humans, but for other *Helicobacter* species the level of pathogenicity, and hence their clinical relevance, is unclear.[127,128] In a study on non-migratory Canada geese in the USA *Helicobacter* spp. were isolated from faeces in approximately 28% of the birds, including *H. anseris* and *H*. *brantae*.[128] A Swedish study found a clear correlation between host species and bacterial species. For example, all *H. canadensis* isolates were retrieved from geese, and none from other bird species or from grazing cattle.[126] Hence, there is currently not enough evidence to assess the possibility of transmission of *Helicobacter* from geese and swans to humans and livestock, but the host specificity probably mitigates the risks.

##### Yersinia

Some bacteria of the genus *Yersinia*, namely *Y. enterocolitica* and *Y. pseudotuberculosis*, are considered pathogenic for animals and humans, symptoms mainly being gastrointestinal illness.[109] Geese have been shown to carry the strain of *Y. enterocolitica* that causes disease in humans, but also several non-pathogenic species and strains. For example, Niskanen and co-workers [129] found *Yersina* spp. in 42 out of 105 faecal samples from barnacle geese, but none in brent geese, Canada geese, greylag geese, or mute swans (seven, one, one and one samples respectively). Many of these were, however, non-pathogenic species of *Yersinia*, or non-pathogenic strains of *Y. enterocolitica*. The authors commented that as these barnacle geese were sampled on migration they had most likely become infected at a previous location, and then acted as long-distance dispersers. Furthermore, they concluded that, because of the low prevalence of pathogenic strains isolated, birds – including geese – are not likely to be a direct source of *Yersinia* infections in humans.[129]

##### Clostridium botulinum/avian botulism

Botulism, caused by neurotoxins produced by the bacterium *Clostridium botulinum*, affects a wide range of birds and mammals, including humans. The symptoms typically include paralysis. The disease is found globally and is usually acquired via oral intake of the toxin, or – especially in birds – intestinal growth of the bacteria, which then produce toxins in the guts. Different strains of *C. botulinum* produce different toxins, usually referred to as types A through G. Avian botulism is considered the most significant disease of migratory waterfowl in North America,[130] and is caused by other strains than human botulism.

Avian botulism has been known for more than a century. For example, Hay and co-workers already in 1973 published a paper on botulism type C in wild spur-winged geese *Plectropterus gambensis* in South Africa, where they traced descriptions of this disease back to 1893. There are also indications of botulism being present in North America as early as in 1890.[130] In Spain, outbreaks in waterfowl are mainly seen in the warmer seasons, i.e. summer and autumn, due to the combination of high temperatures, large amounts of biomass, and anaerobic conditions in wetlands.[131] To our knowledge, botulism has not been reported as a significant problem in swans and geese (albeit in other types of wild birds, such as gulls) in Northern Europe, and no link between waterfowl and outbreaks in domestic poultry has been established here. For mammals including humans, botulism is linked to consumption of feed or food containing the neurotoxin, i.e. not to contact with birds, which are affected by a different strain. Hence, the risk of wild geese and swans transmitting botulism to humans is negligible, and the risk of transfer to poultry appears limited, and should not warrant any specific action.

##### Brachyspira

The genus *Brachyspira* includes a number of spirochetal bacteria that are pathogenic to birds and mammals including humans, but also some non-pathogenic species. The best known, *Brachyspira hyodysenteriae*, is an important gastrointestinal pathogen in pigs (swine dysentery) and not found in birds. Recently also *Brachyspira hampsonii*, which is another pathogen in pigs, has been found in wild greylag geese (Spain, 4.9%, *n =* 205 [132]). The authors concluded that attention should be paid to the possibility of disease transmission in case of outdoor pig production, with possible contact between wild bird reservoirs of *Brachyspira* spp. More research is needed to assess the actual risk from this pathogen as spread by geese or swans. *B. hampsonii* is not known to be pathogenic to humans.

#### Respiratory

##### Chlamydophila psittaci/chlamydiosis, ornithosis or psittacosis

Avian chlamydiosis is caused by the bacterium *Chlamydophila psittaci* (previously *Chlamydia psittaci*), which causes mild to severe illness in both birds and mammals, including humans. Zoonotic transfer is linked to inhalation or direct contact.[133] The disease is sometimes referred to as ‘psittacosis’ or ‘ornithosis’, depending on the species affected. Birds are the natural reservoir, and transmission of disease between mammals is rare. Hence, wild birds are considered the source of any case of chlamydiosis in domestic poultry or other domestic animals, and in humans.[134]

Most research on *C. psittaci* has focused on psittacine birds, i.e. parrots, but there is also some information about its occurrence in geese. Dickx and co-workers [133] found *C. psittaci* antibodies in 94% (*n =* 81) of the feral Canada geese sampled in Belgium, and managed to isolate viable *C. psittaci* from 58% (*n =* 47) of these birds, although none of them showed any clinical signs of disease. This can be indicative of a persistent infection. The authors concluded that Canada geese are indeed part of the avian reservoir host system for this bacterium, and that they hence pose a risk to native wildlife.[133] However, a German study analysing eggs (*n =* 289) from Canada geese did not find any *Chlamydophila/Chlamydia* bacteria at all.[24] To summarize, geese can harbour the *Chlamydia* and *Chlamydophila* bacteria, but there is no evidence that they are a relevant source of infection in poultry, livestock or humans.

#### Systemic

##### Borrelia/Lyme disease and borreliosis

*Borrelia* are spirochetal bacteria often divided into groups of different pathogenicity. The best known is *B. burgdorferi*, causing the tick-borne Lyme disease in humans, with symptoms such as fever, severe headache, rash, and joint pain.[135] *B. burgdorferi* has been identified in ticks sampled from a variety of host species including passerine birds.[136] Geese may act as tick population amplifiers and transporters, but the infection is not reported to be common in geese,[135] and it is not considered pathogenic to birds. It is extremely unlikely that transfer from geese would be of any relevance to mammals, including humans.

The disease caused by *Borrelia anserina* (avian spirochetosis) in birds is characterized by acute septicaemia, and commonly seen in domestic poultry in tropical and subtropical regions,[135] transmitted by *Argas* spp. ticks.[137] It has never been reported in wild birds and does not affect humans,[135] and it is mainly controlled by improved vertical integration and biosecurity within the poultry industry in affected regions.

#### Antimicrobial resistance

Resistance to one or more pharmaceutical antibiotic substances is not an infectious disease *per se*, but it is often treated as such for practical reasons as they pose large problems in human and animal health. There are a number of different types of resistance mechanisms, which will however not be covered in detail here. Use and misuse of antibiotics, in humans, domestic animals and agriculture, are the main drivers in the development of antibiotic resistance.[138,139] It is generally acknowledged that antibiotic resistance is a rapidly emerging threat to human health, causing millions of deaths every year as a result of failure to treat common infections.[140] The prevalence of antibiotic resistance varies considerably among countries and continents,[141,142] much depending on whether use of antibiotics is prudent or not.

Geese, and other birds, may become colonized by antibiotic resistant bacteria just like they pick up other bacteria present in their environment. It has hence been hypothesized that birds can act as amplifiers or vectors, carrying these bacteria and in the end transmitting them to livestock via pastures and to humans by contamination of human food or water sources.[143] Several studies on the prevalence of various bacteria in geese include analysis of faecal samples with reference to antibiotic resistance.

In their paper on *Brachyspira hampsonii*, Martinez-Lobo and co-workers [132] studied the antibiotic susceptibility of their *B. hampsonii* isolates (*n =* 10) from Spain. They found that all isolates were susceptible to the lowest antibiotic concentration tested, and hence there was no indication of resistance in these bacteria.

Conversely, Middleton and Ambrose,[144] who analysed *E. coli* in Canada geese in the USA, found that more than 95% of their isolates (*n =* 47) was resistant to a variety of antibiotic substances, such as ampicillin, cephalothin, and sulfathiazole. Fallacara and co-workers,[95] who studied Canada geese and other waterfowl in the USA, identified antibiotic resistance in some of the *E. coli* and *C. jejuni* strains in their samples (specific figures for goose samples not presented). Another US study focusing on non-migratory Canada geese found very different levels of antibiotic resistance in *E. coli* isolated from faeces, depending on the type of agricultural land the geese inhabited, in relation to livestock manure. The authors reported low or zero levels of resistance in samples from geese in no contact with liquid livestock manure, whereas geese in direct contact with liquid swine manure had a significantly higher prevalence of antimicrobial resistance.[143] A Japanese study on *E. coli* in Whistling swans *Cygnus columbianus* also revealed a high prevalence of antibiotic resistance (279 *E. coli* isolates from 984 swans, of which 80% (*n =* 244) of the drug resistant isolates showed resistance to more than one antibiotic).[145] Similarly, Hatha and co-workers,[146] who sampled barnacle geese (*n =* 30) at breeding sites in Svalbard, found 100% resistance to colistin in their *E-coli* isolates, modest levels of resistance to ampicillin (39%) and amoxicillin (12%), and low levels against tetracycline (7%) and ceftazidime (2%), but the bacteria were still susceptible to a number of other antibiotic substances. Birds in this study winter in the UK, and the authors speculated that antibiotic resistance genes may have been picked up in that environment, rather than in the more pristine Svalbard. Recently, Kuczkowski and co-workers [107] found a higher prevalence of antibiotic resistant *E. coli* strains in birds sampled in Poland compared to geese in the Netherlands, and hypothesized that this may be a result of difference in proximity to human dwellings. However, also other explanations are possible, such as the general level of antimicrobial resistance in the countries in question, and also in the regions where these goose populations spend time during breeding and migration.

To summarize, waterfowl spending time in areas close to human waste or domestic animal manure containing high levels of bacteria carrying antibiotic resistance are likely to pick these up, and can act as vectors for such bacteria. However, compared to other sources and transfer possibilities of antimicrobial resistance, the importance of this particular pathway is probably limited. Moreover, as the interpretation of resistance is dependent on the methodology used, future studies should to a higher extent combine phenotypic measures with characterization of the underlying molecular mechanisms of resistance, including sequencing of genes and plasmids carrying resistance markers.

### Unicellular endoparasites

Among the unicellular endoparasites, three groups are of interest in geese and swans, *Cryptosporidium, Giardia*, and Microsporidia (Table 1).

#### Cryptosporidium

Sixteen species of *Cryptosporidium* are widely acknowledged among nearly 50 genotypes found, with several species described rather recently as a result of new genetic differentiation methods.[147,148] These methods are still under development, and some of the early *Cryptosporidium* studies are therefore less useful. Some species seem to be adapted to a single host species, while others appear to be found in several. The systematics of *Cryptosporidium* are still debated, and Feng et al. [149] point out the uncertainty level also with present methods. They state that although closely related genetically, even small differences between genotypes can influence host specificity. Studies performed only at the generic level, i.e. *Cryptosporidium* sp., have therefore been excluded from the present review (for example [150] on Canada goose, and [151] on greylag goose and bean goose).

Species considered as common human pathogens are *Cryptosporidium hominis, C. parvum, C. meleagridis, C. felis*, and *C. canis.*[148] Symptoms in humans include diarrhoea and abdominal pain, and humans are most commonly infected via contaminated water.

The main route suggested for disease transmission is contamination of food or water by manure from cattle.[148] The role of geese in transmission of *Cryptosporidium* to humans seems to be limited, Kassa et al. [152] even stating that no known cases exist. If Canada geese have a role, it would probably be as a mechanical vector only, for *C. hominis* and *C. parvum.*[148,153–155] An experiment by Graczyk et al. [156] showed that when Canada geese were inoculated with *C. parvum* oocysts no infection seemed to occur; rather all oocysts were excreted through faeces. The Canada goose itself is infected by two isolated genotypes that are called ‘goose genotypes I and II’, which do not affect livestock or humans.[148,149,153]

The transmission routes where geese can act as carriers start either when they pick undigested corn in faeces from cattle [157] or when they are contaminated by humans visiting their feeding area.[153] Then geese could visit and later contaminate either drinking reservoirs or human recreational areas.[153]

Due to the uncertainties regarding methods and nomenclature, the zoonotic potential of geese and swans to either livestock or humans is not possible to evaluate for the *Cryptosporidium* species. Still, there might be a small risk of transmission from birds consuming contaminated feed and passing undigested *Cryptosporidium* to freshwater reserves, which are used by livestock and humans for drinking.

#### Giardia

*Giardia duodenalis* (synonyms *G. lamblia* and *G. intestinalis*), is a flagellate protozoan that may contaminate water as well as food. It causes diarrhoea in humans, and it may also affect growth and cognitive function in children.[158]

In Poland, *Giardia duodenalis* has been found in greylag geese (prevalence 29%; *n =* 34), mute swans (12%; *n =* 33), and domestic geese (9.1%; *n =* 11).[155] It has also been found in greylag geese and domestic geese in Hungary.[151] *Giardia* sp. has been found in Canada geese in the USA [150,157] and in bean geese in Hungary.[151] Some studies have reported high concentrations, which might indicate infection in these birds, while Majewska et al. [155] reported lower concentrations, suggesting that those birds were merely mechanical vectors.

All studies cited above were carried out before new methodology revealed that there are at least eight genotypes or assemblages, named A through H, that have the same morphological features. The host specificities for these eight genotypes are currently considered to be humans and other vertebrates (A, B), dogs (C, D), hoofed livestock (E), cats (F), rats (G) and seals (H).[158] This insight into the genetics of *Giardia* was highlighted by Plutzer and Tomor [151] as a limitation to their study.

Due to high prevalence of *Giardia*, geese and swans become suspects of transferring the pathogen to humans, but this potential risk is still not well understood. Further studies based on the newest taxonomical knowledge are needed to establish whether *Giardia* in geese belong to genotype A or B, thus being capable of causing disease in humans.

#### Microsporidia

Human microsporidiosis occurs mainly in immunosuppressed persons and leads to intestinal infections with diarrhoea. Microsporidia is a wide taxonomic grouping of unicellular parasites comprising thousands of species, of which at least 14 can infect humans.[159] Among the latter, *Encephalitozoon hellem* and *Encephalitozoon intestinalis* have been found in geese, and these are known to cause most cases of microsporidiosis in humans. Microsporidia occur in surface water and may survive up to a year at low temperatures.[160] In Poland, *Encephalitozoon hellem* was found in greylag geese (prevalence 9%, *n =* 34), mute swans (13%, *n =* 30), and in captive swans of three species (25–100%; *n =* 1–4), whereas *Encephalitozoon intestinalis* was found in domestic geese (9%, *n =* 11).[160]

The suggested transmission pathway to humans is from faeces from geese and swans. However, this has never been demonstrated and the actual connection between occurrence in birds and human disease is thus unclear. The potential risk for transfer to humans and livestock is through surface water used for drinking water or recreation.

## Discussion

### Knowledge gaps and quality of research

The research about zoonotic diseases in geese and swans relevant to livestock fairly well mirrors which species occur close to agriculture and man. For example, in the review text above there are 17 mentions of studies concerning greylag geese, 16 of mute swans, eight of greater white-fronted, and eight of barnacle geese. In line with the species distribution, all but one study on mute swans are from NW Europe (one is from the USA, where the species is introduced). However, the most frequently mentioned species in our review is Canada goose (44 mentions), which is widespread in NW Europe as well as in its native North America, often occurring abundantly close to man. Most studies on this species were carried out in North America, but are obviously relevant also for European conditions. From a NW European perspective, bean goose, pink-footed goose, and whooper swan, on the other hand, remain understudied considering that they are numerous and usually occur in agricultural areas during the non-breeding season.

A tabulation by sampling season did not indicate any seasonal bias in the studies on which the present review is based. Somewhat fewer studies were based on birds sampled in spring than in other seasons, but admittedly spring is the shortest season from the perspectives of climate, as well as in the annual cycle of these birds.

A problem in a review like this is the variety of methodologies used for sampling, storage and screening in the analysed articles. This affects both the sensitivities of assays, such as serology, cultivation or molecular detection, and the level of characterization. Regarding the latter, the advancement of sequence-based methods now allows for multi-gene or genome comparisons, providing a much more detailed level for assessing occurrence of particular traits, such as virulence markers or resistance mechanisms.

This is especially evident in e.g. *E. coli, Campylobacter, Giardia*, and *Cryptosporidium*. Another concern is that so many studies are based on faecal samples collected in the field (i.e. not on cloacal swabs). This is problematic because there may be uncertainty as to which species produced the dropping sampled, and because there is always an issue of contamination from bacteria already present on the ground (from other species). Care is needed when designing future surveillance studies, as to safeguard the highest possible turnout.

### Potential transmission pathways

Interestingly, many of the studies reviewed here have identified both potentially pathogenic and non-pathogenic microbes in geese and swans even though the sampled birds did not show any signs of disease. This is partly because not all microbes pathogenic to humans or livestock are necessarily pathogenic to geese and swans, but also because the occurrence of clinical disease is always related both to the presence of a given pathogen and to various characteristics of the host and the environment. For instance, susceptibility and disease severity are likely linked to the condition of the bird, which can be exacerbated by local weather and temperature. Birds can also, apart from becoming actively infected themselves, act as dispersers of various microbes or microbe-carrying ticks. However, many authors have, in line with Benskin et al. [161], Dieter et al. [162], and Tsiodras et al. [7], emphasized that it is difficult to determine whether or not waterfowl pose significant disease transmission risks to human and/or domestic animal health. By and large, the scientific basis for most of the implied associations between pathogens in waterfowl and disease in humans remains anecdotal or speculative.[7,162] Furthermore, several authors appear reluctant to quantify or downplay risks. For example, a Norwegian study based on 219 faecal samples from greylag geese, five from mallards and 200 from feral pigeons *Columba livia ‘feral’* found very few or no birds positive for *C. jejuni, Salmonella*, paramyxovirus, or avian influenza virus, but still the authors concluded that wild birds may constitute a reservoir for important pathogens and zoonotic agents.[26] Although such a statement may still be true, it avoids putting the true risk into perspective. We argue there is a tendency to regard geese and swans as more problematic as zoonotic agents than evidence warrants. In this context, it should be emphasized that detection of a pathogen in an animal is not the same as the species being a competent reservoir host that allows for forward transmission. This is particularly important when it comes to rare pathogens, where single detections in a host species rather could reflect a spill-over event from an unknown source. Ultimately, results from screening studies should be complemented with experimental studies, targeting key aspects of disease dynamics such as shedding time/length of infection, bacterial/viral loads, pathogenicity and ecological costs of infections.

#### Transmission from geese and swans to poultry or livestock

The potential role of wild birds as source of infectious agents to domestic poultry and livestock is mainly linked to faecal contamination of water supplies, pastures and feed.[161] Indeed, geese (and swans) produce abundant faecal material where they graze and roost,[4] but this does not have any, or only a very short-lived, negative effect on the attractiveness of grasslands to livestock (review in [3]). In principle this sets the scene for transfer of pathogens from wild geese and swans via water and pasture-land to livestock, but the importance of this pathway is still far from understood. It is also difficult to provide general answers to questions about potential health risks related to geese and swans, as the risks will vary considerably not only within a region (climate and density of birds, poultry, and livestock), but also with the type of domestic animal husbandry prevailing. Regardless, several studies stress the importance of proper on-farm biosecurity and disease surveillance systems (see for example [163]). This is a rather intuitive conclusion, and in fact such biosecurity precautions have been in place for poultry for decades in NW Europe and other regions. In other words, the absence of reports about disease transmission from swans and geese to domestic poultry may be an effect of already existing biosecurity routines. Nevertheless it is wise to apply precautionary principles and continue to ensure that domestic poultry do not get in contact with, or share pasture or water access with, wild waterfowl. For livestock, however, the situation is clearly different, as the presence of swans and especially geese on grasslands and pasture grounds is high in many areas of NW Europe. Still, reports of transmission from swans and geese to livestock are virtually absent, and hence their presence does most likely currently not constitute a major risk factor for livestock in relation to the diseases presented in this review.

Based on the present review, the overall risk of disease transmission from geese and swans to poultry and livestock appears to be comparatively low in NW Europe. In this context it is important to underline that migratory birds may be involved also in dispersal of pathogens to more distant and new geographical locations. This, in turn, may indirectly increase the risk of disease transmission, for example when a pathogen is brought to an environment that is more benign for it,[7] or the pathogen is introduced to another species that in turn has a higher risk of transmitting it to domestic animals (e.g. ‘bridge species’ for AIV).

#### Transmission from geese and swans to humans, directly or via wild birds, poultry or livestock

The main possible transmission routes are direct contact between geese and swans and humans (beaches, parks), indirect contact (faecal material) and via the human consumption of grain products and vegetables, or milk, meat or eggs from infected domestic animals. In an extensive general review, Tsiodras et al. [7] concluded that many wild bird species can serve as reservoirs or vectors for a number of different pathogens, but they found only one case of direct transmission from wild geese and swans to humans (a cluster of HPAI H5N1 infections in people plucking feathers from dead mute swans), and also relatively little evidence for indirect transmission. Overall, they argued that direct transmission from wild birds in general play a limited role in human infectious diseases.[7] They also emphasized the importance of effective public information campaigns to put perceived risk into perspective, stressing that public activities in areas with abundant wild birds carry minimal risk, in particular if people refrain from possible risk activities such as unprotected handling of dead, wild waterfowl. In this context it is worth noting that several European countries now allow or even promote intensified goose culling (e.g. [164]), which would increase direct contact between geese and humans.

Although wild geese and swans, based on present knowledge, have a very limited role in zoonotic disease transmission, we have noticed that publications directed towards the public or staff involved in managing geese tend to emphasize rather than downplay disease transmission risks.[165,166] Although sometimes stated that such transmission is not well understood or documented, a general advice is nevertheless to minimize direct contact between humans and goose faeces.[166] Furthermore, Abulreesh et al. [167] argued that skin contact and accidental ingestion of contaminated water from amenity village ponds harbouring waterfowl should be avoided, and Gorham and Lee [5] concluded that as Canada geese can indeed be an important source of faecal contamination of recreational waters, population control of this species may be considered for public health reasons.

#### Transmission of pathogens from livestock, poultry or humans to geese and swans

Disease transmission between wild birds and livestock and humans is often seen as a one-directional pathway, but in reality it is bi-directional.[161,164] One obvious and well-documented example is when geese share water or pasture with livestock and poultry, and pick up pathogens from these domestic animals.[143,157] Furthermore, sewage works ponds, which may harbour high numbers of human pathogens, often attract wild waterfowl. In the scope of the present review, the possibly most significant pathogens that may spill back from humans or domestic animals into nature and infect wild birds are HPAIV and *Salmonella*. Modern techniques allowing strain typing show that many of the pathogens found in faeces from waterfowl actually originate from human or domestic animal sewage or slurry or other types of human waste.[153,161] Whatever is present in the environment, the birds may pick up; however, an important question to ask is how well they may serve as reservoirs once transmission has occurred. For instance, Girdwood et al. [86] showed that gulls in Scotland frequently were infected with *Salmonella* originating from human waste activities (7.8% of 5888 samples), but that the duration of shedding was short (four days) and the bacterial load was estimated to be too low to allow further transmission to cattle on pastureland. Similar studies are lacking for most geese and swan pathogens, with the exception of HPAIV.

Occurrence of antibiotic resistant bacteria in wild geese and swans is another example of how pathogens from humans and livestock spill back into nature, and where wild birds potentially can spread these bacteria over longer distance. It is not surprising to find that there are large differences between, for example, North American and European populations of geese when it comes to prevalence of antibiotic resistant bacteria,[132,143,144] as the use of antibiotics in animal production is less restrictive on the former continent.

### Management implications from a One Health perspective

#### General assessment

The potential for zoonotic disease outbreaks linked to migratory birds, including geese, is often much exaggerated in media, which may influence public perception of these birds. Also, some of the studies assessed in this review seem to overestimate the potential risk of geese (e.g. [5]), compared to our conclusions, and to regard them as a ‘source’, rather than possible links in a transmission chain or a circle. Other authors have found that when exploring ecosystems and looking into global health risks, e.g. avian influenza, in relation to industrial scale animal production, there is a tendency to overrate the importance of wildlife.[168] Nevertheless, the wildlife–livestock interface in disease ecology must not be neglected and warrants continued attention, which is exemplified by the recent incursion of HPAIV H5N8 and reassortant viruses in North America.[63] Our present synthesis demonstrates that swans and geese can be colonized/infected by a number of microorganisms that can cause disease in humans and/or domestic animals, although for some of these microbes there are uncertainties about their host-specificity. However, there is very little evidence of such transmission actually taking place, and we therefore argue that although there are potential risks, these are often exaggerated in line with the precautionary principle. This may divert attention from more relevant risk factors.

An explicit aim of the present review is to assess the risk of disease transmission from wild herbivorous anatids (swans, geese) to livestock, poultry, and humans. Based on the research available, we accordingly classify the pathogens treated here into three basic groups of concern in a NW European context:

1. Present evidence suggest that geese and swans may play a role, although minor, in transmission of avian influenza virus, *Salmonella, Campylobacter*, and antibiotic resistant bacteria.
2. There is no present evidence that geese and swans play a role in transmission of Newcastle disease, duck plague, West Nile virus, *Vibrio, Yersinia, Clostridium, Chlamydophila*, and *Borrelia*.
3. Data deficient – based on present knowledge it is not possible to say if geese and swans play a role in transmission of *E. coli, Pasteurella, Helicobacter, Brachyspira, Cryptosporidium, Giardia*, and Microsporidia.

It is worth underlining that our analysis does not point out any pathogen at all in which geese and swans play a truly significant role for zoonotic spread compared to other wildlife, at least not from a European perspective. Most probably, our grouping above also holds for the situation in North America, due to the fact that several studies were performed there and that many conditions and species are the same. From a One Health perspective, this risk of disease spread must be weighed against positive contributions by swans and geese, for example as ecosystem engineers, biodiversity, and other ecosystem services.[169]

We would like to point out that this review focuses on specific disease transmission only. This means that we have not evaluated other possible goose- and swan-related risks to human and animal health. Food poisoning from eating infected goose meat or infections acquired during hunting and carcass dressing have, for example, not been dealt with. Neither have we included feed quality issues related to heavy faecal soiling of ley fields. In this case it has been proposed that if the proportion of goose faecal material is very high and especially where the feed is not completely dried but stored semi-dried (silage and haylage, often for horse fodder), general quality problems may occur due to e.g. formation of mould or spores in the bales.

#### Management advice

As concluded above, we find less evidence to worry about disease transmission by geese and swans than is often the case, also from a One Health perspective. There is a tendency to overrate negative effects of geese in disease transmission as in other areas, for example crop damage and wetland eutrophication.[3,4] We nevertheless advocate the following:

1. A large share of the pathogens treated in this review is spread via water and the faecal–oral pathway. It is a wise precaution to keep poultry and livestock separated from ponds and other small volume waters used by wild waterfowl. This advice must be used wisely and should not hinder use of cattle to graze for example nature reserves, also along shores. When livestock have access to lakes or ponds where large numbers of geese and swans stage or reside, it is advised to supply water to domestic animals in other ways, such as troughs or bowls.
2. There is no need to prevent livestock from grazing on pastures where geese and swans are present.
3. In areas where there is a risk of contamination of freshwater reservoirs and other potential lakes for use of freshwater, deliberate and systematic scaring of birds may be used to encourage them to choose other lakes as roosting places.
4. For recreational lakes there is a small but not negligible risk of spread of disease both from humans to waterfowl and from waterfowl to humans. In recreational areas where geese are abundant, swimmers should be encouraged to avoid swallowing water, and proper sanitary facilities for humans and dogs should be provided.
5. Normal hygiene procedures are important for people who handle wild birds, and especially for those who also get in contact with poultry and livestock. For staff involved in very sensitive poultry production, such as the daily management of valuable breeding stock, both keeping of backyard poultry or pet birds and direct contact with wild geese and swans should be avoided.
6. For a limited number of rare (from a European perspective) but quite devastating diseases, such as avian influenza and West Nile virus (fever), and possibly also anatid herpesvirus, continuous monitoring sampling of wild birds should be encouraged. However, geese and swans are not necessarily the most appropriate focal species for such sampling schemes. The choice of species must be carefully considered and evaluated before such activities are launched at a larger scale.
7. As a contribution to minimizing the development of and slowing the spread of antimicrobial resistance, prudent use of antibiotics in humans and animals is necessary at the global scale, and more effective anti-bacterial control is desired at wastewater ponds used by swans, geese and other waterfowl. Where this cannot be met, discouraging waterfowl from breeding or staging there may be considered.
8. It should be kept in mind that knowledge and disease situations are prone to change. With future increasing knowledge about these pathogens, with new or emerging diseases becoming more (or possibly less) common, or with changes in human behaviour or in farm animal husbandry systems, there may be reasons for regularly reviewing these recommendations and adapting them to situations that may be different from the ones we see today.

Research needs from a One Health perspective:

1. Taxonomic: There is an urgent need to develop (and use existing) methods to better understand taxonomy and host specificity of several pathogens. This is especially true for *E. coli, Campylobacter, Giardia* and Microsporidia. Also, research should be prioritized about the pathogens for which present knowledge is particularly insufficient to evaluate the role of geese and swans in their transmission (e.g. *E. coli, Brachyspira, Cryptosporidium*, and Microsporidia).
2. Transmission pathways: To be efficient from a One Health and disease prevention perspective, future research should focus on finding out if (and if so, when and where) actual transmission between wild waterfowl, domestic farm animals and humans, and vice versa, does occur. Modern genotyping methods may play an important role in establishing if host-specificity is present, and at what levels, and is a helpful tool in source attribution efforts.
3. Counter-measures: From this review it is clear that geese and swans are not a major source of infections in humans or domestic animals for any of the diseases covered. However, geese and swans are, as many other types of wildlife, part of the total circulation of various microbes, of which some are pathogenic to humans, livestock or poultry. In order to prevent unnecessary disease in any of these categories, the pattern of transfer of these pathogens need to be better understood.
4. Relative role of geese and swans versus other wild birds: It is obvious in some cases (e.g. avian influenza) that geese and swans may differ from dabbling ducks when it comes to susceptibility, and thereby in their role as reservoirs and transmitters. Since many geese occur closer to humans and agriculture than most dabbling ducks, such differences warrant further study. Until then, great caution must be taken when making inferences about geese and swans based on knowledge related to other types of waterfowl.
5. Pathogenicity: Field-based studies should be complemented by increased number of experimental infection studies to better quantify disease progression, bacterial/viral load, and risks for forward transmission.
6. Weighing of risks and benefits: Disease prevention is certainly an important aspect of One Health, but the presence and preservation of wetlands, waterfowl and related factors also have important environmental and recreational benefits. Hence, further studies are needed to evaluate risks of disease transmission in relation to other important aspects, such as ecosystem values of waterfowl and their habitats.
